# Short- and Long-Term Mortalities of Small and Large Larvae of *Alphitobius diaperinus* (Panzer) (Coleoptera: Tenebrionidae) on Concrete Surfaces Treated with Three Insecticides: Impact of Food

**DOI:** 10.3390/insects13040366

**Published:** 2022-04-08

**Authors:** Nickolas G. Kavallieratos, Erifili P. Nika, Anna Skourti, Constantin S. Filintas, Theofania D. Goumenou

**Affiliations:** Laboratory of Agricultural Zoology and Entomology, Department of Crop Science, Agricultural University of Athens, 75 Iera Odos Str., 11855 Athens, Attica, Greece; erifilinika@aua.gr (E.P.N.); annaskourti@aua.gr (A.S.); stud216085@aua.gr (C.S.F.); stud217021@aua.gr (T.D.G.)

**Keywords:** lesser mealworm, piperonyl butoxide+acetamiprid+d-tetramethrin, etofenprox, deltamethrin, poultry

## Abstract

**Simple Summary:**

In this study, we used etofenprox, deltamethrin, and the combination of piperonyl butoxide+acetamiprid+d-tetramethrin as surface treatments on concrete (with or without food) to evaluate the short- and long-term mortalities of the lesser mealworm, *Alphitobius diaperinus* (Panzer) (Coleoptera: Tenebrionidae) small and large larvae. Concerning short-term mortality, etofenprox killed 97.8% and 80.0% of the small and large larvae, respectively, 7 days post-exposure on concrete without food. Deltamethrin caused complete (100.0%) mortality to small larvae without food (3 days post-exposure), small larvae with food, and large larvae without food (5 days post-exposure), and 98.9% mortality to large larvae with food after 5 days of exposure. Piperonyl butoxide+acetamiprid+d-tetramethrin killed all small larvae without food 5 days post-exposure. Concerning long-term mortality, all small larvae exposed to etofenprox died on concrete without food, while piperonyl butoxide+acetamiprid+d-tetramethrin caused 85.0% mortality to small larvae on concrete with food. Overall, deltamethrin was the most efficient active ingredient for the management of both small and large larvae of *A. diaperinus*.

**Abstract:**

The lesser mealworm, *Alphitobius diaperinus* (Panzer) (Coleoptera: Tenebrionidae) is an important stored-product pest for the poultry industry as it is a vector of dangerous pathogens for humans. In the present study, we evaluated the short- and long-term mortalities of small and large larvae of *A. diaperinus* when they were exposed to concrete-covered Petri dishes treated with etofenprox, deltamethrin, and the combination of piperonyl butoxide+acetamiprid+d-tetramethrin. Small and large larvae were exposed to each insecticide applied on concrete surfaces with or without food. The short-term mortality was recorded after 1 day, 3 days, 5 days, and 7 days, while the long-term mortality was recorded 7 days after the transport of the larvae alive to pesticide-free concrete-covered dishes. Regarding short-term mortality levels, 97.8% and 80.0% of the small and large larvae, that were exposed to etofenprox without food, died after 7 days of exposure, respectively. Concerning deltamethrin, all tested small larvae were killed after 3 days (without food) and 5 days (with food) of exposure. For large larvae, deltamethrin caused 98.9% (with food) and 100.0% (without food) mortality levels after 5 days of exposure. The combination of piperonyl butoxide+acetamiprid+d-tetramethrin caused high mortality levels to small larvae, i.e., 84.4% and 100.0% on dishes with and without food, respectively, but low to moderate mortality levels to large larvae that did not exceed 67.8% after 7 days of exposure. Long-term mortality varied vastly among the tested insecticides. Etofenprox killed 100.0% of the small larvae on concrete without food, but 24.0% of the large larvae exposed to concrete containing food. Deltamethrin did not provide long-term mortality to large larvae when food was present. Piperonyl butoxide+acetamiprid+d-tetramethrin caused mortality rates that overall varied from 33.5% (large larvae on concrete with food) to 85.0% (small larvae on concrete with food). In conclusion, deltamethrin killed almost all exposed larvae at exposures of ≤5 days, regardless of their size and the presence of food on the concrete.

## 1. Introduction

The lesser mealworm, *Alphitobius diaperinus* (Panzer) (Coleoptera: Tenebrionidae), is a secondary pest of stored products and a scavenger that occurs globally [1,2]. It infests 77 commodities such as nuts, cereals, vegetables, tobacco, chocolate, and animal products [2,3]. It has been observed eating dead birds and mice [1,4]. Usually, it can be found at henhouses, poultry houses, stables, grain bins, mills, and warehouses [2,3]. Adults are black, oval, and 5–7 mm long [1,2,5], while larvae are brown, elateriform, and approximately 15 mm long [1]. This species is of major importance concerning public health, since both adult and larvae are vectors of multiple pathogens such as *Salmonella enterica* ssp. *enterica* (ex Kauffmann and Edwards, 1952) Le Minor and Popoff, 1987 serovars Enteritidis and Typhimurium (Enterobacterales: Enterobacteriaceae), and *Campylobacter jejuni* (Jones et al., 1931) Veron and Chatelain, 1973 (Campylobacterales: Campylobacteraceae) [6,7]. These pathogens can cause severe symptoms to humans such as diarrhea, bloody diarrhea, fever, abdominal cramps, and vomiting [8,9,10]. Interestingly, *A. diaperinus* larvae transmit *S. enterica* ssp. *enterica* serovar Enteritidis more efficiently than the adults, as chicks that consumed infested larvae had higher bacterial concentrations on their spleen, liver, and cecum than chicks that consumed infected adults [11]. Therefore, the effective management of this species, especially at poultry houses, is highly important.

The larvae of *A. diaperinus* have become resistant to several chemical insecticides over time [12,13,14] due to their continuous use. Kaufman et al. [15] found that tetrachlorvinphos 500 g/kg sprayed on plywood panels killed only 55.7% of the exposed larvae. Later, Chernaki-Leffer et al. [16] documented the resistance of *A. diaperinus* to cypermethrin, dichlorvos, and triflumuron. Approximately 208,136 ng cypermethrin/cm^2^, 179,366 ng dichlorvos/cm^2^, and 1534 ng triflumuron/cm^2^ were needed to kill 90% of the exposed *A. diaperinus* population. Furthermore, 8265.5 ppm cypermethrin dust was needed to kill 90% of *A. diaperinus* larvae [17]. When larvae were exposed to wood shavings treated with the label dose of cypermethrin dust (0.0573 g/cm^2^), mortality levels did not exceed 50% [17]. After 7 days of exposure to a treated poultry bedding with an insecticidal combination of citronellal, cypermethrin, and chlorpyrifos, 59.26% of larvae were killed [18]. A recent study showed that alpha cypermethrin, spinosad, and pirimiphos methyl were not effective against *A. diaperinus* larvae [19]. The management of *A. diaperinus* larvae faces difficulties when alternative insecticides such as essential oils (EOs) are used [20]. For example, the northern white cedar, *Thuja occidentalis* L. (Pinales: Cupressaceae), and the common tansy, *Tanacetum vulgare* L. (Asterales: Asteraceae) EOs, as well as their main constituents, i.e., α-thujone and *β*-thujone, caused low mortality levels to *A. diaperinus* larvae, not exceeding 30% [20]. Tomasi et al. [21] reported that 0.4 mg/mL glycerol monolaurate killed <30% of the exposed larvae in Petri dishes containing treated filter papers, 8 days post-exposure.

Etofenprox [2-(4-ethoxyphenyl)-2-methylpropyl-3-phenoxybenzyl ether] (supercooled liquid) is a pyrethroid insecticide that affects the neurotransmission through the prolonged activation of the sodium channels [22]. It is characterized as an ingestion and contact insecticide with light stability and over 3 months half-life [22,23,24]. Deltamethrin [(s)-ca-cyano-m-phenoxybenzyl(1R,3R)-3-(2,2-dibromovinyl)-2,2-dimethylcyclo-propane-carboxylate] is also a pyrethroid insecticide that has a similar mode of action with etofenprox, by causing prolonged activation of the neuronal membranes’ sodium channels [25]. Piperonyl butoxide (3,4-methylenedioxy-6-propylbenxyl n-butyl diethyleneglycol ether) is an aliphatic-aromatic polyether that acts as an insecticide synergist [26]. It has long stability (over 18 years), enhances the insecticidal properties of other insecticides, and inhibits the detoxification activity of the insects by reducing the cytochrome P450 levels [26,27,28]. Acetamiprid [N-[(6-chloropyridin-3-yl)methyl]-N′-cyano-N-methylethanimidamide] is a neonicotinoid insecticide that acts on the nicotinic acetylcholine receptors (nAChR) of insects, resulting in their excitation, paralysis, and death [29]. Lastly, d-tetramethrin [3,4,5,6-tetrahydrophthalimidomethyl (1RS)-cis-trans-chrysanthemate] is a pyrethroid insecticide with a similar mode of action to etofenprox and deltamethrin, i.e., it attacks the insects’ nervous system by acting on the function of sodium channels [23,30,31,32].

Even though many insecticidal formulations have been tested against *A. diaperinus* larvae, etofenprox, deltamethrin, and the combination of piperonyl butoxide+acetamiprid+d-tetramethrin have never been evaluated as surface treatments for the management of this species. After a meticulous search of the published literature, only the combination of piperonyl butoxide+chlorpyrifos has been previously tested in contact toxicity assays [33]. Therefore, the objectives of the current study were to evaluate the short- and long-term mortalities of etofenprox, deltamethrin, and the combination of piperonyl butoxide+acetamiprid+d-tetramethrin on concrete against small and large larvae of *A. diaperinus*.

## 2. Materials and Methods

### 2.1. Insects and Food

Coleopteran individuals were cultured at 30 °C, 55% relative humidity, and in total darkness [34,35] at the Laboratory of Agricultural Zoology and Entomology, Agricultural University of Athens. The founding individuals were obtained from a laboratory colony that was kept in the Laboratory of Agricultural Entomology (Benaki Phytopathological Institute, Kifissia, Attica, Greece). For the experimentation, unsexed small larvae (<7 mm) and large larvae (>7 mm) were used [1]. The rearing medium was wheat bran (75%) and yeast (25%), with apple cuttings for extra moisture [35]. The rearing medium was used as food in the experiments. Diet moisture was 12.3%, as determined by a calibrated moisture meter (mini GAC plus, Dickey-John Europe S.A.S., Colombes, France).

### 2.2. Formulations

The formulations that were used in the current experiments were: Phobi E EC with 300 gr/l etofenprox active ingredient (a.i.) (provided by Protecta, Peristeri, Attica, Greece), K-Othrine WG with 25% deltamethrin (a.i.) (provided by Bayer Hellas, Amaroussion, Attica, Greece), and Dobol^®^ EC with 10% piperonyl butoxide, 5% *w*/*v* acetamiprid and 2.5% *w*/*v* d-tetramethrin (a.i.) (provided by Société Kwizda France, Marly le Roi, France).

### 2.3. Bioassays

The formulations were applied at commercially labeled doses for surface applications. Consequently, etofenprox was tested at 0.015 mgr (a.i.)/cm^2^, deltamethrin at 0.0025 mg (a.i.)/cm^2^, and piperonyl butoxide+acetamiprid+d-tetramethrin at 0.0002 mL formulation/cm^2^. For the experiment, we prepared three replicates (with each replicate having 3 subreplicates) of Petri dishes (8 cm diameter and 1.5 cm high). Each subreplicate was constituted by 10 dishes, containing one individual each, to ensure that the insects will not cannibalize each other [36]. The surface area of each dish was 50.27 cm^2^. The bottoms of the dishes were covered by concrete (CEM I 52.5 N material, Durostick, Aspropyrgos, Attica, Greece) 24 h before the beginning of the experiments. The lids of all the dishes had a hole (1.5 cm diameter) covered with gauze to aerate the internal space of the dishes. Polytetrafluoroethylene (60 wt% dispersion in water) (Sigma-Aldrich Chemie GmbH, Taufkirchen, Germany) was applied to the inside vertical part of the dishes to ensure the enclosure of the tested individuals in the dishes. Each concrete-covered dish was treated with an aqueous solution of total volume of 1 mL with the proper concentration of each tested formulation. The fine mist was achieved by using a separate airbrush (AG-4 Mecafer S.A., Valence, France) for each formulation. Thereupon, we weighted 0.5 g quantities of the diet with an electronic balance (Precisa XB3200D, Alpha Analytical Instruments, Gerakas, Attica, Greece) that were transferred into each concrete-covered dish. Additional series of dishes were prepared with the exact same procedure, but diet was not added. Furthermore, extra series of dishes were produced as controls, with or without food, but this time they were sprayed with distilled water with a different airbrush. In each prepared dish a single small or large larva was inserted. Then, the dishes were put into incubators set at 30 °C and 55% relative humidity and total darkness. The mortality of both small and large larvae was counted under a stereomicroscope (Olympus SZX9, Bacacos S.A., Athens, Greece) with 57× magnification, 1 day, 3 days, 5 days, and 7 days post-exposure, with a fine brush (Cotman 111 No 000, Winsor and Newton, London, UK) by nudging gently the tested individuals. If no movement was detected, individuals were considered dead. The individuals that were alive were transferred to new untreated concrete-covered dishes after 7 days of exposure, to evaluate their long-term mortality. For the larvae that had food into the dishes, new quantities of 0.5 g diet were put into the new untreated dishes. After 7 days, the dead individuals were also counted as described above.

### 2.4. Data Analysis

Short- and long-term mortalities of the controls was low (<5%), consequently no correction was applied to the data. Prior to the analysis, data were log (x + 1) converted to normalize the variance [37,38]. The repeated-measures model was used to run the analysis, separately for each larval size group [39]. In the analysis, exposure was the repeated factor. Mortality was the response variance. Insecticide and presence/absence of food were the main effects. In the analysis, the associated interactions of the main effects were considered. Data for long-term mortality were submitted to a two-way ANOVA, separately for each size of larval group. Mortality was the response variable. Insecticide and the presence/absence of food were the main effects. Means were separated using the Tukey-Kramer honest significant difference (HSD) test at the 0.05 significance level [40]. The JMP 14 software was used for all analyses [41].

## 3. Results

### 3.1. Short- and Long-Term Mortalities of Alphitobius Diaperinus Small Larvae

All main effects and their associated interactions, between and within exposure intervals, were significant (Table 1). Etofenprox caused elevated short-term mortality to *A. diaperinus* small larvae (97.8%) after 7 days of exposure without food (Table 2). When food was present on concrete, mortality rates were significantly lower than when food was absent at all exposure intervals, not exceeding 64.4% at the end of the experimental period. Complete mortality (100.0%) was achieved when concrete was treated with deltamethrin without containing food after 3 days of exposure and after 5 days of exposure on concrete when food was existed (Table 3). After 1 day of exposure, mortality levels were not significantly different with or without food (46.7% and 75.6% mortality, respectively). Concerning piperonyl butoxide+acetamiprid+d-tetramethrin, complete mortality was achieved when food was absent, that differed significantly when food existed (60.0%), 5 days post-exposure (Table 4). Yet, when food was present, 84.4% of the exposed larvae were dead, after 7 days of exposure. Long-term mortality was moderate on concrete treated with etofenprox with the presence of food (64.1%) and complete on concrete without food (100.0%), but they were not statistically different (Table 5). Piperonyl butoxide+acetamiprid+d-tetramethrin killed 85.0% of the remaining individuals on concrete with food at the end of the experimental period.

### 3.2. Short- and Long-Term Mortalities of Alphitobius Diaperinus Large Larvae

All main effects and their associated interactions were significant between and within exposure intervals (Table 1). Regarding short-term mortality, etofenprox killed significantly fewer exposed individuals (37.8%) with the presence of food than without food (80.0%), 7 days post exposure (Table 2). Complete mortality was documented on concrete treated with deltamethrin without food while 98.9% was the achieved mortality on concrete with food after 5 days of exposure (Table 3). Piperonyl butoxide+acetamiprid+d-tetramethrin caused low to moderate mortality at large larvae, not exceeding 13.3% (on concrete with food) and 67.8% (on concrete without food), that was significantly higher, 7 days post-exposure (Table 4). Concerning long-term mortality, etofenprox resulted in low (24.0%) and moderate (53.7%) mortality levels, with and without food, respectively (Table 5). No long-term mortality was recorded on concrete treated with deltamethrin with the presence of food. Significantly more large larvae died on concrete without food (68.2%) than on concrete with food (33.5%) in the case of piperonyl butoxide+acetamiprid+d-tetramethrin.

## 4. Discussion

The results of this study have revealed that etofenprox, deltamethrin, and the combination of piperonyl butoxide+acetamiprid+d-tetramethrin are effective against both small and large larvae of *A. diaperinus*, with or without food. Previous knowledge has revealed that larvae of *A. diaperinus* cannot be easily managed. For example, in a field study, Hess et al. [42] examined six insecticidal treatments against larvae of this species. They found low reduction of the larval individuals at five out of the six scenarios. Steelman [43], by applying 450.41 mg/g cyfluthrin or 489.75 mg/g methoxychlor to the prosternum of each larva, caused 50% mortality. To achieve 90.0% of larval mortality, 112.6 ppm cyfluthrin or 132.0 ppm of the imidacloprid were needed [44]. After 7 days of exposure to dishes covered with a biocidal paint containing ultramarine+permethrin+violet 23, 41.33% of the larvae were killed, while 82.67% mortality was achieved 14 days post-exposure [45]. Similarly, filter paper treated with 1% EO, 1% EO-nanoemulsion (NE), and 1% EO-nanocapsules (NC) of the true cinnamon tree, *Cinnamomum verum* Jan Svatopluk Presl (J.Presl) [= *Cinnamomum zeylanicum* Blume] (Laurales: Lauraceae), caused approximately 50%, 40%, and 20% mortality, respectively, after 7 days of exposure [46]. In a recent study, the mortality caused by pirimiphos-methyl, spinosad, and alpha-cypermethrin against *A. diaperinus* larvae exposed to treated concrete did not exceed 40.0%, with or without food [19].

The strain of the exposed *A. diaperinus* individuals should be taken into account since different strains are more or less susceptible to certain insecticides. Cyfluthrin, permethrin, permethrin+acetamiprid, and pirimiphos-methyl were examined against twelve *A. diaperinus* strains [47]. Among these strains, three of them exhibited pyrethroid resistance, while five of them were susceptible to all insecticides. According to Hichmann et al. [48], nine geographical strains of this species exhibited variable resistance ratios ranging between 1.0-fold and 17.0-fold (cypermethrin) and between 1.5-fold and 20.5-fold (chlorpyrifos). Due to the above resistance issues identified on several *A. diaperinus* strains, multiple a.i. could be an efficient way to manage this species [49].

At all experiments on concrete surfaces with food, mortality levels were lower than when food was absent, for small and large larvae, for both short- and long-term mortalities. This observation indicates that the presence of food plays an important role for the effectiveness of a.i. because food degrades and/or absorbs part of the treated insecticides, acting as a barrier between the treated surface and the tested individuals. In previous experiments, the efficacy of methoprene alpha-cypermethrin, chlorfenapyr, spinosad, deltamethrin, pirimiphos-methyl, cyfluthrin, and pyriproxyfen decreased when food was present on concrete surfaces [19,50,51,52,53,54]. Moreover, food can impede insects from contacting the treated concrete surfaces, increasing the percentage of the surviving individuals [50], and can also absorb a part of the insecticide, which could lead to the death of the feeding insect individuals [55]. Toews et al. [56] reported that the increased amount of food on the treated plastic surfaces resulted in a decrease in the mortality rate of the red flour beetle, *Tribolium castaneum* (Herbst) (Coleoptera: Tenebrionidae). Our findings showed that deltamethrin was highly effective either with the presence or the absence of food. This is an important finding since previous studies have documented that the presence of food on deltamethrin treated surfaces can moderate the efficacy of this insecticide. For example, Velki et al. [57] found that for 10%, 50% and 90% *T. castaneum* mortality, higher doses of deltamethin were needed at treatments with flour than at treatments without flour. Similarly, Boukouvala and Kavallieratos [54] showed that more khapra beetle, *Trogoderma granarium* Everts (Coleoptera: Dermestidae) eggs hatched on treated concrete surfaces with food than on without food. Additionally, mortality levels were higher on concrete surfaces without food. Apparently, the performance of deltamethrin depends on the exposed species when surface treatments is followed as a management strategy. Whether *A. diaperinus* adults are subject to the same pattern merits further investigation.

Our results reveal that small larvae were more susceptible than large larvae. This issue could be attributed to the fact that different age larvae have cuticle with various thickness, softness and epicuticular lipid composition [58,59,60,61]. Additionally, large larvae are less vigorous than small larvae since they are close to becoming pupae [60], and therefore move less on the treated surface leading to lower mortality levels. In a historic study, Gast [62] found that the more a larva weighs the more insecticide is needed to kill it. Since small and large larvae are exposed to the same dose of toxicant, it is expected that small larvae will die more easily. This trend has been previously documented by several researchers [61,63,64]. Small *A. diaperinus* larvae reached 85.0% mortality, while only 7.4% of large larvae were killed by 12.5 mg/mL fruit extract of the star anise, *Illicum verum* Hook. F. (Austrobaileyales: Schisandraceae), 5 days post-exposure [65]. Kavallieratos et al. [61] noticed that small larvae of the larger mealworm, *Tenebrio molitor* L. (Coleoptera: Tenebrionidae), were more susceptible than large larvae when they were exposed to three types of grains treated with deltamethrin, pirimiphos-methyl, silicoSec, and spinosad, at all exposure intervals. Similarly, Kavallieratos et al. [63] and Arthur et al. [64] showed that large larvae of *T. granarium* were harder to kill than small larvae when they were exposed to surfaces treated with pirimiphos-methyl, chlorfenapyr, deltamethrin, spinosad, pyriproxyfen, and cyfluthrin. The combination of piperonyl butoxide+acetamiprid+d-tetramethrin was highly effective against small larvae of *T. granarium* on concrete, although there are no comparative data with large larvae, since 84.4% of the exposed individuals died 7 days post-treatment at 0.0002 mL formulation/cm^2^ [66].

## 5. Conclusions

To conclude, the current study sheds light on the efficacy of etofenprox, deltamethrin, and the combination of piperonyl butoxide+acetamiprid+d-tetramethrin against *A. diaperinus* small and large larvae on treated concrete surfaces with the presence or absence of food, under short exposure intervals. The most efficient insecticide was deltamethrin as it killed almost all exposed small and large larvae, 5 days post-exposure on concrete with or without food. Etofenprox and piperonyl butoxide+acetamiprid+d-tetramethrin caused elevated short- and long-term mortality levels, but they depend on the presence or absence of food and the size of the larvae. Further experimentation is needed to fully unravel the potentials of the tested insecticides applied on different types of surfaces, e.g., different types of storage, plastic, wood, tile, on several food commodities, and against different developmental stages of *A. diaperinus*.

## Figures and Tables

**Table 1:** MANOVA parameters for main effects and associated interactions for mortality of *Alphitobius diaperinus* small and large larvae between and within exposure intervals (error DF = 48).

Effect		Small Larvae		Large Larvae	
Between exposure intervals					
Source	DF	*F*	*p*	*F*	*p*
Intercept	1	4233.7	<0.01	2939.7	<0.01
Insecticide	2	30.8	<0.01	170.5	<0.01
Food	1	35.1	<0.01	169.7	<0.01
Insecticide × food	2	8.6	<0.01	30.8	<0.01
Within exposure intervals					
Exposure	3	89.1	<0.01	222.9	<0.01
Exposure × insecticide	6	19.1	<0.01	18.2	<0.01
Exposure × food	3	18.1	<0.01	8.4	<0.01
Exposure × insecticide × food	6	4.4	<0.01	12.0	<0.01

**Table 2:** Mean short-term mortality (% ± SE) of *Alphitobius diaperinus* small and large larvae exposed to concrete treated with etofenprox for 1 day, 3 days, 5 days, and 7 days. Within each row, means followed by the same uppercase letter are not significantly different (in all cases DF = 3, 35, Tukey-Kramer HSD test at *p* = 0.05). Within each column, means that are followed by the same lower-case letter are not significantly different (in all cases DF = 3, 35, Tukey-Kramer HSD test at *p* = 0.05).

Larval Group	1 Day	3 Days	5 Days	7 Days	*F*	*p*
Small larvae (with food)	5.6 ± 1.8 Cb	18.9 ± 5.1 BCb	28.9 ± 4.2 ABb	64.4 ± 8.4 Ab	11.0	<0.01
Small larvae (without food)	25.6 ± 1.8 Ca	57.8 ± 4.9 Ba	82.2 ± 4.7 Aa	97.8 ± 1.5 Aa	97.5	<0.01
Large larvae (with food)	0.0 ± 0.0 Cc	1.1 ± 1.1 Cc	14.4 ± 2.4 Bc	37.8 ± 5.2 Ac	61.7	<0.01
Large larvae (without food)	3.3 ± 1.7 Dbc	13.3 ± 1.7 Cb	35.6 ± 1.8 Bb	80.0 ± 2.4 Aab	54.9	<0.01
*F*	22.5	24.0	18.1	20.8		
*p*	<0.01	<0.01	<0.01	<0.01		

**Table 3:** Mean short-term mortality (% ± SE) of *Alphitobius diaperinus* small and large larvae exposed to wheat treated with deltamethrin for 1 day, 3 days, 5 days, and 7 days. Within each row, means followed by the same uppercase letter are not significantly different (in all cases DF = 3, 35, Tukey-Kramer HSD test at *p* = 0.05). Within each column, means that are followed by the same lower-case letter are not significantly different (in all cases DF = 3, 35, Tukey-Kramer HSD test at *p* = 0.05). Where no letters exist, no significant differences were recorded.

Larval Group	1 Day	3 Days	5 Days	7 Days	*F*	*p*
Small larvae (with food)	46.7 ± 5.8 Ba	98.9 ± 1.1 Aa	100.0 ± 0.0 A	100.0 ± 0.0 A	20.6	<0.01
Small larvae (without food)	75.6 ± 5.3 Ba	100.0 ± 0.0 Aa	100.0 ± 0.0 A	100.0 ± 0.0 A	17.2	<0.01
Large larvae (with food)	1.1 ± 1.1 Bb	62.2 ± 5.7 Ab	98.9 ± 1.1 A	98.9 ± 1.1 A	224.7	<0.01
Large larvae (without food)	7.8 ± 3.6 Bb	64.4 ± 5.0 Ab	100.0 ± 0.0 A	100.0 ± 0.0 A	40.0	<0.01
*F*	41.4	21.8	1.0	1.0		
*p*	<0.01	<0.01	0.41	0.41		

**Table 4:** Mean short-term mortality (% ± SE) of *Alphitobius diaperinus* small and large larvae exposed to wheat treated with piperonyl butoxide+acetamiprid+d-tetramethrin for 1 day, 3 days, 5 days, and 7 days. Within each row, means followed by the same uppercase letter are not significantly different (in all cases DF = 3, 35, Tukey-Kramer HSD test at *p* = 0.05). Within each column, means that are followed by the same lower-case letter are not significantly different (in all cases DF = 3, 35, Tukey-Kramer HSD test at *p* = 0.05).

Larval Group	1 Day	3 Days	5 Days	7 Days	*F*	*p*
Small larvae (with food)	5.6 ± 2.4 Bb	36.7 ± 5.3 Aa	60.0 ± 7.6 Ab	84.4 ± 5.6 Aa	34.7	<0.01
Small larvae (without food)	12.2 ± 2.2 Ba	58.9 ± 6.6 Aa	100.0 ± 0.0 Aa	100.0 ± 0.0 Aa	41.4	<0.01
Large larvae (with food)	0.0 ± 0.0 Bc	0.0 ± 0.0 Bc	0.0 ± 0.0 Bd	13.3 ± 3.3 Ab	25.8	<0.01
Large larvae (without food)	0.0 ± 0.0 Cc	11.1 ± 2.0 Bb	37.8 ± 4.7 Ac	67.8 ± 4.0 Aa	131.2	<0.01
*F*	16.6	103.9	547.9	25.8		
*p*	<0.01	<0.01	<0.01	<0.01		

**Table 5:** Mean long-term mortality (% ± SE) of *Alphitobius diaperinus* small and large larvae exposed for 7 days on untreated concrete dishes, with or without food, after 7 days of exposure on concrete dishes treated with etofenprox, deltamethrin and piperonyl butoxide+acetamiprid+d-tetramethrin. Within each row, asterisks indicate significant differences (two-tailed *t*-test at *p* = 0.05). Within each column, means that are followed by the same lower-case letter are not significantly different (Tukey-Kramer HSD test at *p* = 0.05). Where no letters or no asterisks exist, no significant differences were recorded. Where dashes exist, no analysis was performed.

Inscecticide	Small Larvae					Large Larvae				
	Food	No Food	DF	*t*	*p*	Food	No Food	DF	*t*	*p*
Etofenprox	64.1 ± 9.6	100.0 ± 0.0	8	1.4	0.20	24.0 ± 6.4 ab	53.7 ± 12.0	17	0.8	0.43
Deltamethrin	-	-	-	-	-	0.0 ± 0.0 b	-	-	-	-
Piperonyl butoxide+ acetamiprid+ d-tetramethrin	85.0 ± 9.6	-	-	-	-	33.5 ± 2.3 a	68.2 ± 8.4 *	17	4.2	<0.01
DF	12	-				2, 18	17			
*t*	−1.5	-					−1.4			
*F*		-				5.2				
*p*	0.15	-				0.02	0.18			

## Data Availability

Data are contained within the article.
